# Workflow to Select Functional Promoter DNA Baits and Screen Arrayed Gene Libraries in Yeast

**DOI:** 10.1002/cpz1.70059

**Published:** 2024-11-21

**Authors:** Iris Fañanás‐Pueyo, Ana‐Mariya Anhel, Ángel Goñi‐Moreno, Luis Oñate‐Sánchez, Gerardo Carrera‐Castaño

**Affiliations:** ^1^ Centro de Biotecnología y Genómica de Plantas, Universidad Politécnica de Madrid (UPM) ‐ Instituto Nacional de Investigación y Tecnología Agraria y Alimentaria (INIA/CSIC), Campus de Montegancedo UPM Pozuelo de Alarcón (Madrid) Madrid Spain; ^2^ Systems Biology Department Centro Nacional de Biotecnologia, CSIC Madrid Spain; ^3^ Departamento de Biotecnología‐Biología Vegetal, Escuela Técnica Superior de Ingeniería Agronómica, Alimentaria y de Biosistemas UPM Madrid Spain

**Keywords:** arrayed libraries, automation, DNA–protein interaction, one‐hybrid system, phylogenetic shadowing

## Abstract

The yeast one‐hybrid system (Y1H) is used extensively to identify DNA–protein interactions. The generation of large collections of open reading frames (ORFs) to be used as prey in screenings is not a bottleneck nowadays and can be carried out in‐house or offered as a service by companies. However, the straightforward use of full gene promoters as baits to identify interacting proteins undermines the accuracy and sensitivity of the assay, especially in the case of multicellular eukaryotes. Therefore, it is paramount to implement procedures for efficient identification of suitable promoter fragments compatible with the Y1H assay. Here, we describe a workflow to identify biologically relevant conserved promoter fragments of *Arabidopsis thaliana* through simple and robust phylogenetic analyses. Additionally, we describe a manual method and its automated robotized version for rapid and efficient high‐throughput Y1H screenings of arrayed ORF libraries with the identified DNA fragments. Moreover, this method can be scaled up or down and used for yeast two‐hybrid screenings to search for possible interactors of proteins identified by the Y1H approach or any other protein of interest, altogether underscoring its suitability to build gene regulatory networks. © 2024 The Author(s). Current Protocols published by Wiley Periodicals LLC.

**Basic Protocol 1**: Selection of DNA baits for Y1H screenings

**Basic Protocol 2**: Y1H screenings with arrayed gene libraries

**Alternate Protocol**: Automated screening with a liquid‐handling robot

## INTRODUCTION

**Figure 1:**
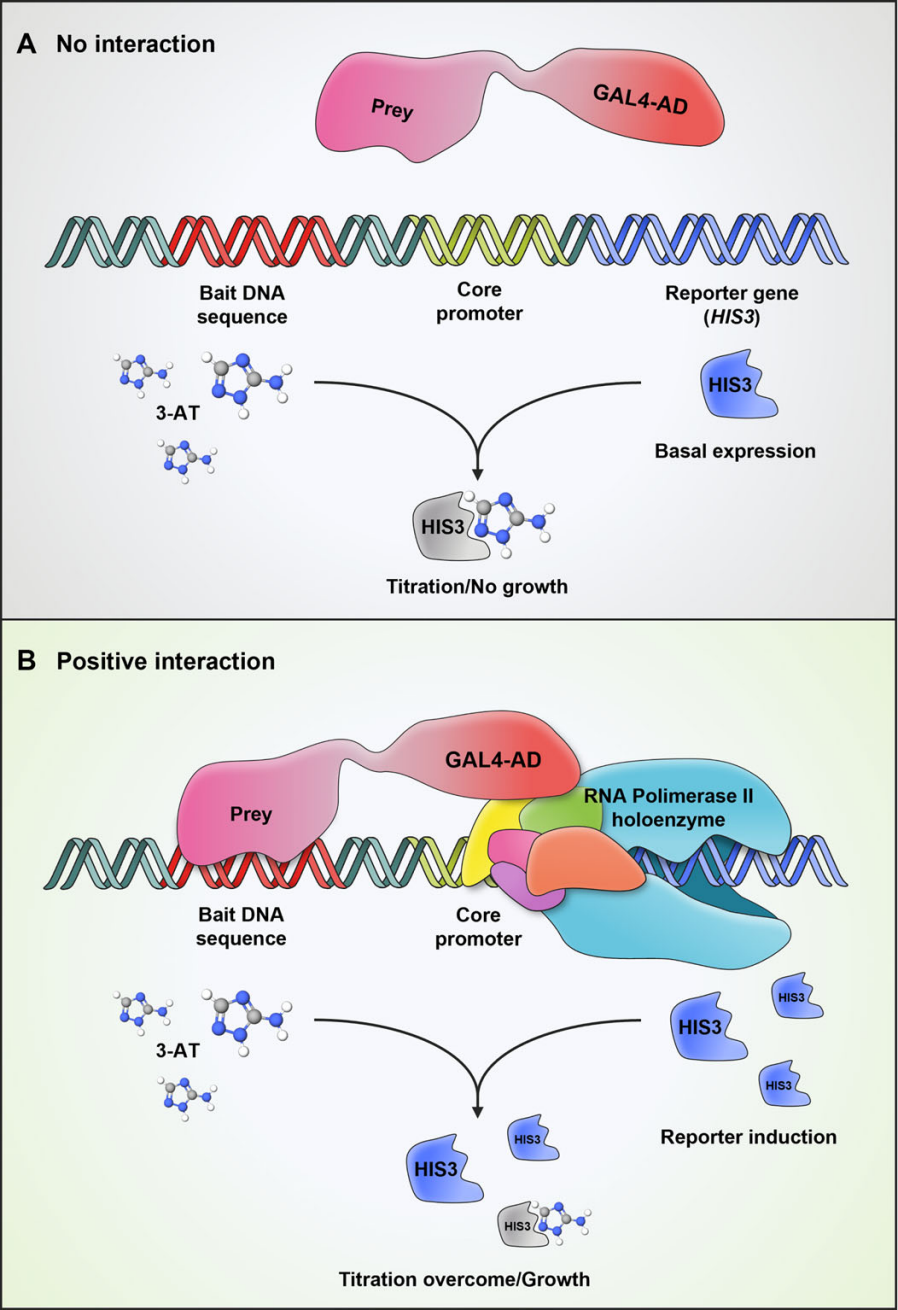
Overview of the yeast one‐hybrid (Y1H) system. (**A**) The bait construct is obtained by cloning a DNA sequence of interest (Bait DNA sequence) upstream of a reporter gene (*HIS3*) carrying a core promoter. The prey construct is obtained by cloning a protein of interest in frame with the GAL4 activation domain (GAL4‐AD). After mating or co‐transformation, although negative control prey(s) should not bind the bait DNA sequence, there might be a basal expression of the reporter gene *HIS3* that can be titrated by the competitive inhibitor 3‐AT. (**B**) If the prey binds to the bait DNA, transcription of the reporter gene *HIS3* will be activated, overcoming the titration with 3‐AT and allowing yeast growth.

Since the original description of the yeast two‐hybrid (Y2H) system to analyze protein interactions (Fields & Song, 1989), several modifications have allowed for an enlargement of the range of molecular interactions that can be analyzed as well as an increase in the throughput (Ferro & Trabalzini, 2013; Ji et al., 2014; Mehla et al., 2015; Mallick et al., 2016; Ota et al., 2014; Reece‐Hoyes & Walhout, 2012; Rezwan & Auerbach, 2012; Snider & Stagljar, 2016). In particular, the yeast one‐hybrid (Y1H) system has been successfully and widely used as a valuable tool to identify transcription factors (TFs) that bind to specific DNA sequences to study eukaryotic gene regulatory networks (Dowell et al., 1994; Inouye et al., 1994; Li & Herskowitz, 1993; Wang & Reed, 1993; Wilson et al., 1991). Transcriptional regulation requires the recognition of short DNA motifs (*cis‐*elements), mostly located in gene promoters, by TF proteins with the ability to modulate their expression levels. With this premise, the Y1H assay was established to couple the specific binding of a TF to the activation of downstream reporter genes, thus leading to the discovery of new DNA–protein interactions. In a classic Y1H assay, two plasmid constructs are used. Typically, one contains the reporter gene under the control of a DNA sequence of interest (bait construct). Another construct (prey) contains the open reading frame (ORF) coding for a protein of interest translationally fused to the ORF encoding the transactivation domain (AD) of the GAL4 TF (GAL4‐AD). The interaction of the prey protein (GAL4‐AD‐prey) with the DNA bait will activate the expression of the reporter gene (Fig. 1). The positive effect of the GAL4‐AD on the transcription of the reporter gene is dominant over the transcriptional properties that the protein of interest may have (i.e., a repressor domain, absence of regulatory domains, etc.), which facilitates the identification of a wider range of interactions (Brent & Ptashne, 1985; Ma & Ptashne, 1998). Several collections of ORFs have been generated and used in Y1H and Y2H screenings in plants and other eukaryotic organisms (Burdo et al., 2014; Brady et al., 2011; Castrillo et al., 2011; Gaudinier et al., 2011; Gong et al., 2004; Mitsuda et al., 2010; Ou et al., 2011; Paz‐Ares J, 2002; Pruneda‐Paz et al., 2014; Taylor‐Teeples et al., 2015). However, methods to identify suitable promoter fragments for efficient and accurate Y1H screenings of these collections are underdeveloped. A common practice is to use full promoters that exceed the size of average yeast promoters, which can significantly reduce or even prevent the activation of the reporter gene as well as increase the number of false positives (Dobi & Winston, 2007; Sánchez‐Montesino & Oñate‐Sánchez, 2018). In Basic Protocol 1, we provide a detailed procedure for the selection and functional evaluation of *bona fide* DNA baits that bolster the efficiency and specificity of the Y1H assay significantly. Then, in Basic Protocol 2, we describe how to use these baits in a simple, rapid, and efficient manual method for high‐throughput Y1H screenings of arrayed ORF libraries. Briefly, mating of sexually compatible strains in liquid media is used to combine bait and prey constructs in the same yeast cells (diploids). After mating, liquid cultures are spotted on two different solid media in parallel to select and score diploid and reporter activation–mediated growth. It only requires approximately 10 h of labor spread over 5 days. Additionally, in the Alternate Protocol, we report how to automate the screening using an Opentrons 2 (OT‐2) robot.

## Basic Protocol 1 SELECTION OF DNA BAITS FOR Y1H SCREENINGS

Basic Protocol 1 describes a series of steps for identifying potential DNA baits for your gene of interest. The identification of DNA baits can be performed by a phylogenetic shadowing approach (Adrian et al., 2010; Boffelli et al., 2003; de Bodt et al., 2006; Hong et al., 2003; Lee et al., 2005; Spensley et al., 2009). This comparative genomic technique, a variant of footprinting analysis, allows the detection of *cis*‐elements by their conservation across multiple evolutionary‐related sequences from orthologous genes within the same family. The assumption is that orthologs exhibit a common regulatory control that is reflected in the conservation of TF binding sites.

### Materials

Oligonucleotides (optional; Table 1)
Plasmids (optional; Table 2)
Online resources:
  Bulk Data Retrieval (https://www.arabidopsis.org/tools/bulk/sequences/index.jsp)
  SeqViewer Nucleotide View (https://seqviewer.arabidopsis.org/servlets/sv)
  Phytozome (https://phytozome.jgi.doe.gov/pz/portal.html)
  JBrowse (https://phytozome‐next.jgi.doe.gov/jbrowse)
  DIALIGN (https://dialign.gobics.de/; Al Ait et al., 2013)
  MEME Suite (https://web.mit.edu/meme_v4.11.4/share/doc/meme.html; Bailey & Elkan, 1994)
  Arabidopsis cis‐regulatory element database (AtcisDB; https://agris‐knowledgebase.org/AtcisDB; Yilmaz et al., 2011)
  AthaMap (http://www.athamap.de/index.php; Steffens et al., 2004)
  Plant Cis‐Acting Regulatory Elements (PlantCARE; http://bioinformatics.psb.ugent.be/webtools/plantcare/html/; Lescot et al., 2002)
  Plant Cis‐acting Regulatory DNA Elements (PLACE; (https://www.dna.affrc.go.jp/PLACE/?action=newplace; Higo et al., 1999)
  The Plant Promoter Analysis Navigator (PlantPAN; (https://plantpan.itps.ncku.edu.tw/plantpan4/index.html; Chow et al., 2024)
  Plant Transcriptional Regulatory Map (PlantRegMap; https://plantregmap.gao‐lab.org/; Tian et al., 2020)
  **Table 1:** List of Primers for Sequencing or PCR Amplification

PRIMERS	SEQUENCE			DESCRIPTION
pTUY1H‐F	5′	CACGAGGCCCTTTCGTCTTC	3′	Forward primer annealing before the *XmaI*/*SmaI* site of the pTUY1H
pTUY1H‐R	5′	TTCTTCGAAGAAATCACATTAC	3′	Reverse primer annealing after the *XbaI* site of the pTUY1H
GAL4AD‐F	5′	TATAACGCGTTTGGAATCACT	3′	Forward primer annealing near the C‐terminal region of the GAL4‐AD in the pDEST22 plasmid
pDEST‐R	5′	AGCCGACAACCTTGATTGGAGAC	3′	Reverse primer annealing downstream of the gateway region in the pDEST22 plasmid (also in pDEST32)
  **Table 2:** Plasmids Used in this Protocol

Plasmid	Cloning	Bacterial selection	Yeast selection	Reference
**pYRO** (transient expression assay in planta)	*BamH1‐Hind3*	Spectinomycin	‐	Thatcher et al., 2007
**pTUY1H** (DNA bait)	*XmaI‐XbaI*	Ampicillin	Leucine (L)	Castrillo et al., 2011
**pDEST22** (protein prey)	Gateway	Ampicillin	Tryptophan (W)	**Invitrogen**

### Orthologue selection

1 Download the promoter sequence corresponding to your *Arabidopsis thaliana* gene of interest from the TAIR webpage using the Bulk Data Retrieval or SeqViewer Nucleotide View tools.
  The selected promoter should include proximal sequences, as most regulatory elements are usually located in the first 300 bases, with a recommended maximum length of 1‐2 kb upstream of the predicted start of each ORF.
2 Download a list of putative Brassicaceae homologues for the *A. thaliana* genes of interest from the Phytozome webpage, as well as their promoter sequences.
  To achieve proper sensitivity, the compiled list is recommended to include at least six members from different Brassicaceae lineages. We have selected the following species from three Brassicaceae lineages: Arabidopsis thaliana (At), Arabidopsis lyrata (Al), Descuriainia sophia (Ds), Carrichtera annua (Ca), Hornungia petraene (Hp), Brassica olereacea (Bo), Thelungiella halophila (Th), and Capsella rubella (Cr). Performing the analysis with a narrow phylogenetic range will not allow accurate determination of conservation, whereas wide phylogenetic distances could overlook shorter candidate motif sequences. When working with plant species other than A. thaliana, you should adapt the analysis to the available resources (see Critical Parameters).
3 Perform a genomic synteny analysis with the JBrowse genome browser tool to evaluate orthologous relationships among the identified homologues.
  High conservation of gene order in both upstream and downstream adjacent regions to the promoter is expected across orthologous genomic regions.

### Motif identification

**Figure 2:**
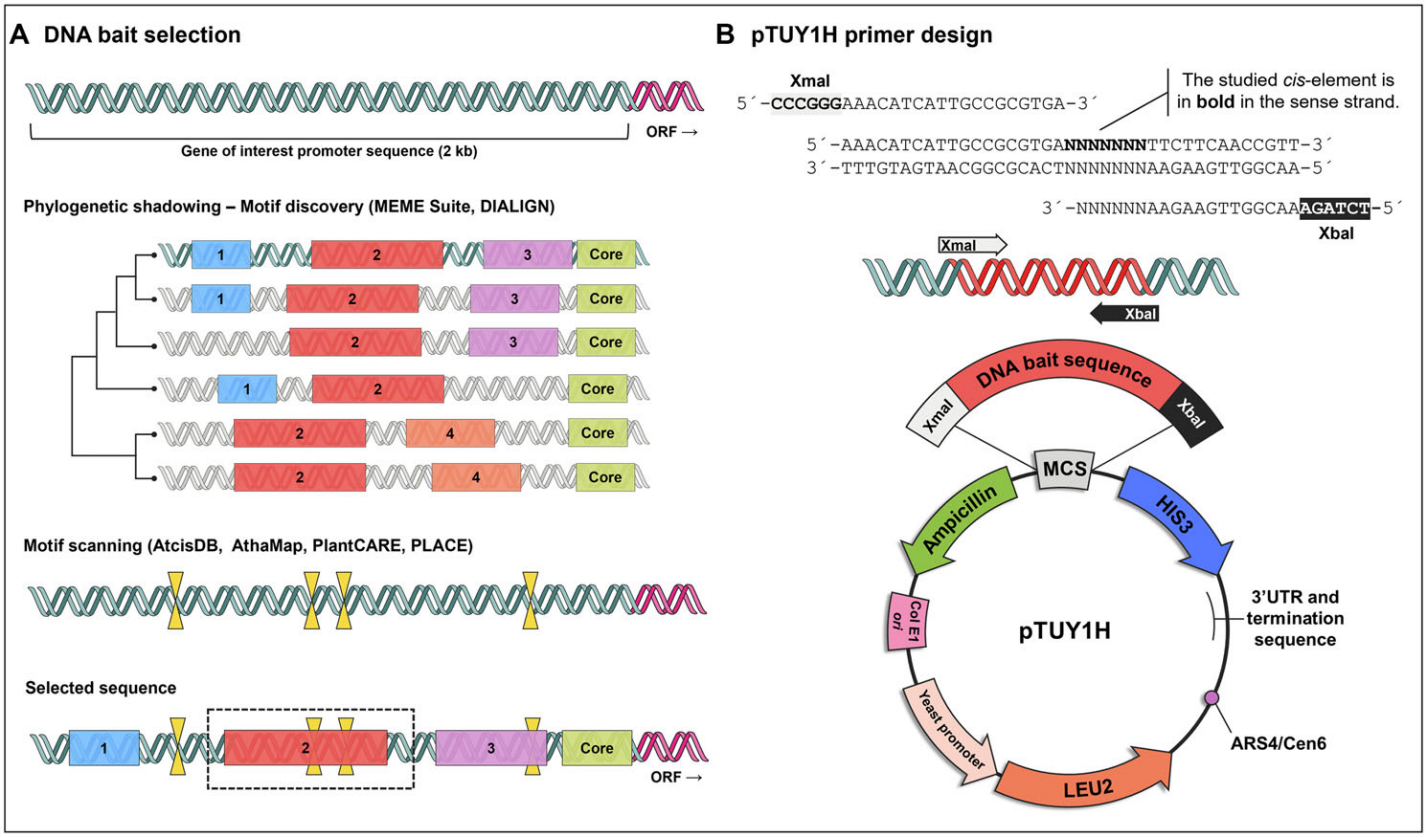
Workflow of *in silico* analyses to select DNA Baits for Y1H screenings. (**A**) DNA bait selection through orthologue analysis and motif identification. (**B**) Graphic scheme of primer design for pTUY1H cloning of the DNA bait using the *HIS3* sequence as an example.

4 Use the final compiled promoter sequences as input for the detection of conserved blocks using the following:
  - Local multiple alignment tools, like the DIALIGN algorithm, count with high resolving power to detect conserved blocks. Its main strength is its ability to discover local homologies among sequences without detectable global homology.
  - MEME suite. MEME discovers novel, ungapped motifs (recurring, fixed‐length patterns) in your sequences. It splits variable‐length patterns into two or more separate motifs.
5 Perform a generic search for consensus TF binding sites using some of the following tools:
  - AtcisDB in AGRIS
  - AthaMap
  - PlantCARE
  - PLACE database
  - PlantPAN
  - PlantRegMap
    Using these databases and tools can be somewhat confusing. It is necessary to curate the data and complement the information through bibliographic searches of known TF binding sites and searching for them in your sequence.
6 Choose one or more promoter fragments based on well‐conserved sequences among species. The presence of already identified *cis*‐elements and known binding sites in those sequences can be used to prioritize them for yeast screenings (Fig. 2A). It is very important to select bait promoter fragments not much longer than 100 bp for Y1H assays (see Critical Parameters).
  A workflow focused on the EXPA2 promoter analysis is described (see Understanding Results).

### Analysis in planta (optional)

Before carrying out the Y1H screening with the selected promoter fragment/s, we recommend performing previous analyses in planta by transient or stable expression to identify whether the chosen fragment may be functionally relevant in the transcriptional regulation of the gene of interest. *Nicotiana benthamiana* can be used for transient expression analyses.

7 Prepare a construct with your bait DNA in a plasmid containing a minimum promoter upstream of a reporter gene.
  We usually use luciferase in the pYRO plasmid (Table 2). If you do not use the pYRO plasmid in transient expression experiments in N. benthamiana, be careful to include a minimum promoter in your construct. Additionally, you can use tandem multimers of the fragment of interest (usually rendering higher expression levels) or a promoter version of your gene of interest with the deleted motif to compare with a non‐mutated version. In addition, other systems could be used such as protoplasts or transient expression in Arabidopsis.
8 Perform a transient expression assay and evaluate the reporter activity (e.g., for luciferase activity, we use a cooled CCD camera or a luminometer).
  There are numerous protocols for agroinfiltration and transient expression assays in N. benthamiana. We usually use the protocol described by Espinosa‐Ruiz et al. (2017).
  Sometimes, a reliable analysis of the bait may require the production of stable transformants given the tissue and/or developmental‐specific nature of the studied promoter (e.g., seed germination; Rombolá‐Caldentey et al., 2014; Sánchez‐Montesino et al., 2019).

## Basic Protocol 2 Y1H SCREENINGS WITH ARRAYED GENE LIBRARIES

Basic Protocol 1 leads to identifying potential Y1H DNA baits of your gene of interest. The next steps include cloning the fragment into the pTUY1H plasmid and transforming yeast, titrating the autoactivation of the bait to establish optimal conditions for scoring positive interactors, screening the bait with an arrayed gene library, and finally confirming the positives. This series of steps is detailed in Basic Protocol 2.

### Materials

Oligonucleotides (Table 1)
Plasmids (Table 2)
Yeast strains (Table 3)
DOB‐leucine (DOB‐L; see recipe)
DOB‐tryptophan (DOB‐W; see recipe)
YPAD medium (see recipe)
Herring sperm DNA (HsDNA; 10 mg/mL; Promega; see recipe)
Yeast transformation solution (PATE solution; see recipe)
Sterile water
DOB‐leucine‐tryptophan (DOB‐L‐W; see recipe)
DOB‐leucine‐tryptophan‐histidine (DOB‐L‐W‐H) (see recipe)
3‐amino‐1,2,4‐triazole 2 M stock solution (3‐AT; Sigma‐Aldrich; see recipe)
Absolute ethanol
Yeast or bacterial plasmid miniprep kit (e.g., Zymoprep yeast plasmid miniprep II, Zymo Research)
Prey ORF library or custom‐made clone collection

**Figure 3:**
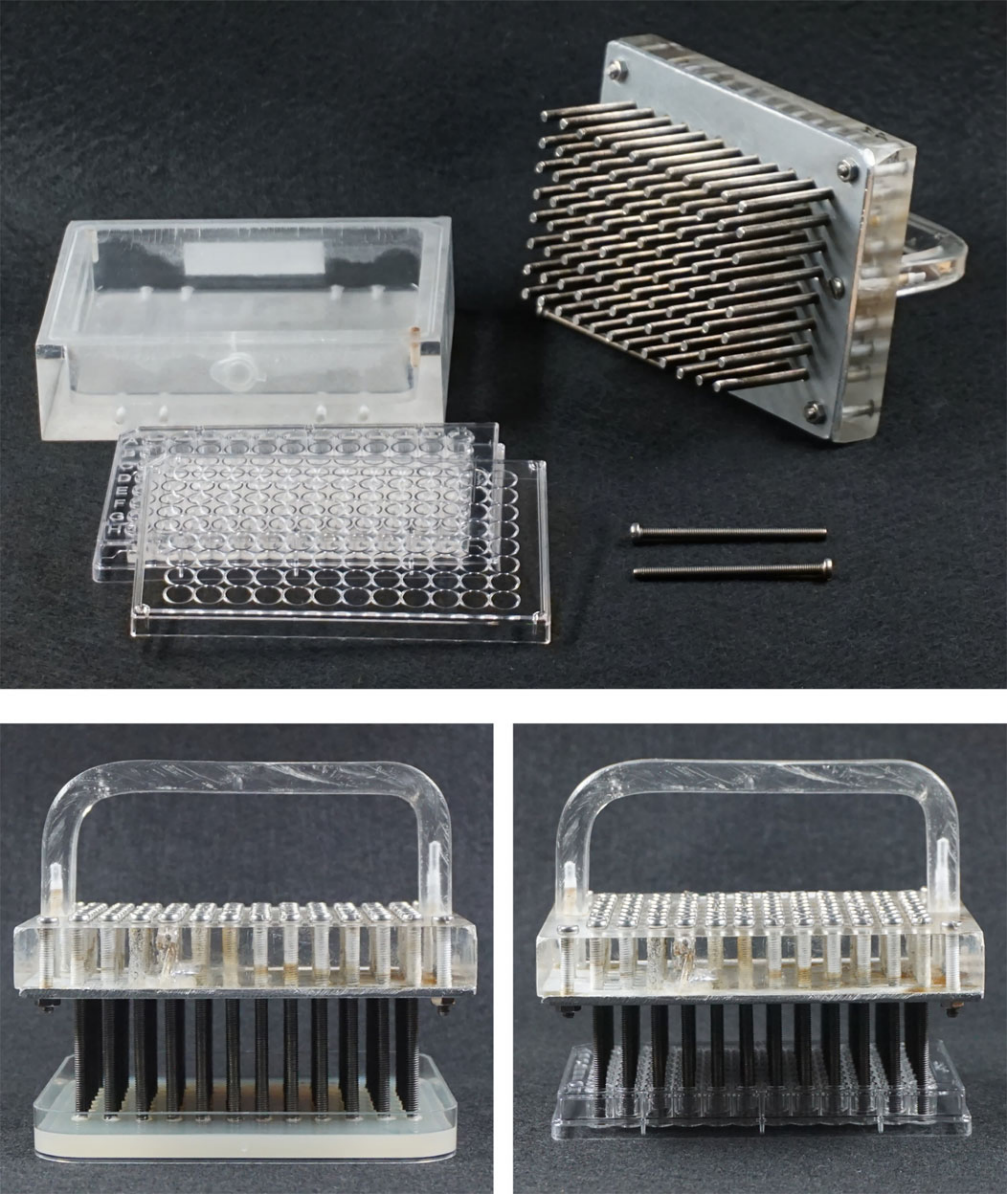
Custom‐made replicator used in our lab. A 3‐mm‐thick stainless‐steel plate (to protect the methacrylate when flaming) was screwed to the bottom of a rectangular methacrylate block (1.8 × 13.3 × 10.1 cm). Then, from the top and through the whole block, stainless‐steel screws (6 cm × 3.5 mm) were screwed in a 96‐well format. For ease of handling, a methacrylate handle was added. This replicator spots ∼5‐10 µL droplets on the agar plates. Any other replicator will do the job.

1.5‐mL Eppendorf tubes
Multichannel pipettes (electronic or manual 12‐ or 8‐channel pipettes to dispense volumes in the range of 100‐250 µL)
10‐mL tubes
Standard shaker or microtiter plate shaker (e.g., HiGro orbital shaker, Gene Machines)
Surgical tape
Parafilm tape
120 mm × 120 mm square petri dishes
96‐well replicator
*We use a custom‐made replicator (* *Fig*. *3* *), but commercial replicators are available (e.g., cat no. R2508, Sigma‐Aldrich; cat. no. 140500, Boekel Scientific; cat. no. 250520, Thermo Fisher Scientific)*.
96‐well plates (standard sterile clear plates with lid and flat bottom; ∼300 µL max. volume/well)
Reagent reservoirs
Erlenmeyer flasks
90‐mm‐diameter petri dishes

**Table 3:** *Saccharomyces cerevisiae* Strain Genotypes

Strain (mating type)	Genotype	Reporters	Auxotrophy	Reference
**YM4271** (**a**)	*MATa, ura3‐52, his3‐200, ade2‐101, ade5, lys2‐801, leu2‐3,112, trp1‐901, tyr1‐501, gal4Δ, gal80Δ, ade5::hisG*		trp1, leu2, his3, ura3 lys2	Liu et al., 1993
**Y187** (**α**)	*MATα, ura3‐52, his3‐200, ade2‐101, trp1‐901, leu2‐3, 112, gal4Δ, met‐, gal80Δ, MEL1, URA3::GAL1UAS‐GAL1TATA‐lacZ*	MEL1 LacZ	trp1, leu2 his3, ade2 met2	Harper et al., 1993

### Preparation of DNA baits for Y1H screenings

1 Generate a construct with your bait DNA sequence in the pTUY1H plasmid (Fig. 2B; Table 2).
  We recommend cloning the DNA sequence of interest in the XmaI and XbaI sites of the pTUY1H plasmid as it will remove most of the multicloning site sequences, which reduces background and distance from the HIS3 start codon. Introduce the construct into S. cerevisiae Y187 (α mating type; Table 3) and select transformants in DOB‐L plates (see Yeast transformation).
  The protocol detailed below can also be used for Y2H screenings (Sánchez‐Montesino & Oñate‐Sánchez, 2018). For this, prepare a construction with your bait protein ORF in the pDEST32 gateway plasmid (Invitrogen). For sequencing or PCR amplification of ORF baits in the pDEST32 plasmid, the oligonucleotides GAL4BD‐Fw (TCATCGGAAGAGAGTAGTAA) or/and pDEST‐R (Table 1) can be used. Introduce this construct (bait) into S. cerevisiae pJ694 (α mating type; James et al., 1996) and select transformants in DOB‐L plates.
2 Prepare a negative control construct for bait titration with the coding sequence of an unlikely protein interactor (i.e., the GFP coding sequence) in the pDEST22 plasmid (Invitrogen; Table 2).
  You will have to introduce this construct (AD‐GFP) and the empty pDEST22 plasmid (AD‐empty) into S. cerevisiae YM4271 (“a” mating type; Table 3) and select transformants in DOB‐W plates (see Yeast transformation). If available, generate a prey strain with the pDEST22 plasmid containing an ORF known to interact with your bait as a positive control.

### Yeast transformation

*This is a modified protocol from that described in* Elble (1992) *. Other yeast transformation protocols are available in the literature, including the preparation of yeast‐competent cells (* *Trimborn et al.*, 2022 *)*.

3 Streak a YPAD plate with the appropriate yeast strain from a frozen stock and incubate for 48‐72 h at 28°C.
4 Inoculate 3 mL of liquid YPAD with a fresh colony and incubate for 24 h at 28°C with shaking.
5 Fill a 1.5‐mL Eppendorf tube with liquid culture containing the 24‐h grown yeast and centrifuge at 3500 rcf for 2 min at room temperature (RT). Discard the supernatant and repeat this step. After the last centrifugation, discard the supernatant by inverting the tube. Resuspend yeast cells in the remaining liquid (∼100 µL total volume); add the following sequentially and mix: i) carrier DNA (usually 5‐10 µL of 10 mg/mL herring sperm DNA); ii) about 1 µg of plasmid DNA; and iii) 500 µL PATE solution. Incubate the tube overnight at RT in darkness (i.e., inside a drawer).
  We do not measure OD, but some protocols indicate that the yeast culture must have OD_600_ ∼ 1.5‐2 when harvested.
  When high colony numbers are not required, the addition of carrier DNA may be omitted. However, we recommend to use it.
  Although 4 h of incubation is enough in many cases, we recommend an overnight incubation.
6 Centrifuge yeast cells at 3500 rcf for 2 min at RT, remove the supernatant with the help of a pipette, and resuspend the cells in 600 µL sterile water by pipetting up and down. Repeat this step twice and finally resuspend the cells in 150 µL sterile water.
7 Plate yeast cells onto appropriate auxotrophic minimal media for positive selection of cells carrying the introduced plasmid. Colonies will appear after 48‐72 h incubation at 28°C.
  The number of colonies is usually highly variable. In a few cases, increasing the starting amount of yeast or the amount of plasmid added is necessary to obtain transformants.

### Titrating bait autoactivation of the HIS3 reporter gene before screening

8 Pick several colonies (5‐20) from transformation plates of i) bait strain, ii) AD‐GFP, and iii) AD‐empty prey strains and streak them onto new plates of the appropriate minimal media. After 24‐48 h incubation at 28°C, use them to inoculate 10‐mL tubes containing 2 mL YPAD medium each. Grow overnight at 28°C with shaking for 24 h.
  It is recommended to secure the caps of the tubes (i.e., with masking tape) as gas produced by yeast fermentation can build up and eject them.
9 Mating: Mix 150 µL of the bait culture with 150 µL of each of the two prey cultures in separate sterile Eppendorf tubes and incubate 24‐72 h at 28°C without shaking.
  Usually, incubation for 24 h is enough to obtain diploids, but we normally incubate 48 h to be on the safe side.
10 Enrichment of diploid cells: Use 100 µL of each of the mated cultures to separately inoculate 900 µL of liquid DOB‐L‐W media in two 10‐mL tubes. Grow at 28°C with shaking for 48‐72 h.
  We routinely incubate for 72 h.
11 Plating diploid cells: Plate 5‐8 µL of each mated culture onto the following agar media: DOB‐L‐W (to quantify diploid cells), and DOB‐L‐W‐H ± 3‐AT (to quantify basal levels of reporter gene activation by the DNA bait). Incubate plates at 28°C and score yeast growth over the next 7 days after plating. Choose the best 3‐AT concentration for screening.
  For the screening plates, we initially use the following range of 3‐AT concentrations (mM): 0, 0.1, 0.25, 0.5, 1, 2.5, 5, 10, 25, and 50. Depending on the information you might have based on previous work with your favorite bait, a different concentration range can be used. It may be necessary to repeat the titration using a tighter range of concentrations to determine precisely the lowest 3‐AT concentration that blocks reporter activation by your bait (after 7 days of incubation) and to be used in the screening.
  This titration protocol can also be used to test one‐to‐one (few) interactions just by including other prey constructs in addition to the controls (i.e., when confirming positive interactions). It also can be used for obtaining a detailed quantification of the strength of the interactions.

### Screening yeast‐arrayed libraries

12 Prior to the following steps, make a fresh replica of the library on DOB‐W square agar plates using a 96‐well replicator and streak one DOB‐L plate with the bait strain. Incubate the plates at 28°C for 48‐72 h (usually 72 h).
  When you use the replicator, sterilize it by flaming it with absolute alcohol. Do not use denatured alcohol to flame the replicator as it contains quaternary amines that will inhibit yeast growth.
  We use this protocol with a library of 15 × 96–well microtiter plates. Adapt media volumes and disposables to your library.
13 Day 1: Using a multichannel pipette, aliquot 125 µL of YPAD medium into each well of 96‐well microtiter plates from a sterile reagent reservoir. Using the replicator, inoculate the 96‐well microtiter plates with the library prey strains grown on the DOB‐W square agar plates. Incubate them at 28°C for 24 h with shaking (250 rpm). In parallel, inoculate an Erlenmeyer containing 12‐15 mL of YPAD/96‐well microtiter plate. For 15 plates, inoculate one 0.5‐1 L Erlenmeyer containing 200 mL of YPAD with a clump of bait cells from the DOB‐L plate and incubate for 24 h at 28°C with shaking (200 rpm).
  To shake the 96‐well microtiter plates, we routinely use a shaker that combines a small shaking orbital (8 mm), gas flow, and temperature controls (2.8 rcf or 250 rpm, 2 s airflow every 30 s, and 28°C). However, in our hands, standard shakers (2.8 rcf) are also amenable to use with this protocol.
  Media and disposables for Day 1: Around 12‐15 mL YPAD medium/96‐well microtiter plate (200 mL for 15 microtiter plates), around 12‐15 mL of YPAD bait culture/96‐well microtiter plate, and one sterile reagent reservoir are needed.
14 Day 2 (mating): Pour YPAD bait culture into a sterile reagent reservoir and using a multichannel pipette, aliquot 100 µL to each well of the 96‐well microtiter plates from Day 1. Incubate for 48 h at 28°C without shaking (weekend incubations are also fine, but we routinely incubate for 48 h).
  Media and disposables for Day 2: One sterile reagent reservoir is required.
15 Day 4 (diploid enrichment): Pour DOB‐L‐W media into a sterile reagent reservoir and using a multichannel pipette, aliquot 200 µL into each well of new 96‐well microtiter plates. Resuspend mated cells in the 96‐well microtiter plates from Day 2 by hitting the bottom of the wells with the pins of the replicator. Then, use the replicator to inoculate the new 96‐well DOB‐L‐W microtiter plates prepared previously. Incubate at 28°C with shaking (2.8 rcf) for 48‐72 h (we routinely wait 72 h).
  Resuspending yeast can sometimes require performing multiple rapid vertical and circular movements with the replicator.
  Media and disposables for Day 4: Around 19.2‐22 mL DOB‐L‐W/96‐ well plate (300 mL for 15 × 96‐well microtiter plates), 15 × 96‐well microtiter plates, and one sterile reagent reservoir are needed.
16 Day 7: Resuspend the cells by hitting the bottom of the wells with the replicator and use each 96‐well DOB‐L‐W microtiter plate to inoculate two square agar plates, one with DOB‐L‐W (diploid plates) and another with DOB‐L‐W‐H ± 3‐AT (selection plates). Once the droplets left by the replicator dry out, close the square agar plates and incubate them at 28°C. Score yeast growth for 7 days.
  Typical results for the EXPA2 promoter screening are described (see Understanding Results).
  This protocol is designed to manually screen libraries arrayed in a 96‐well format, and we have systematically used it with a prey library of ca. 1200 A. thaliana TF ORFs cloned in the pDEST22 plasmid. Diploid (DOB‐L‐W) and screening (DOB‐L‐W‐H ± 3‐AT) plates are inoculated with similar numbers of cells and grown and scored in parallel, allowing eventual non‐mating clones to be flagged as not screened. In any case, diploid colony size and density found after 2 days of incubation of diploid plates should be considered to compare and normalize the strength of positive interactions observed in screening plates.
  Note that fewer diploid cells are typically recovered when mating Y187 with YM4271 in Y1H compared to those obtained between pJ694 and YM4271 in Y2H experiments.
  Media and disposables for Day 7: 15 DOB‐L‐W agar square plates and 15 DOB‐L‐W‐H ± 3‐AT agar square plates
  This protocol is indicated for one bait. The efficiency of the protocol may be enhanced in some of the steps by having two people working simultaneously and by screening two baits at the same time. To do this, prepare twice as many media and disposables. However, some modifications have to be made to screen two independent baits simultaneously:
  For Day 1, aliquot 250 µL (instead of 125 µL) of YPAD into each well of 96‐well microtiter plates.
  For Day 2, by using a multichannel pipette, transfer 100 µL of culture from each well of the microtiter plates from Day 1 to a second set of 96‐well microtiter plates (pipette up and down two or three times to resuspend any settled cells at the bottom of the wells before transferring any liquid to a new plate).
  For Day 6, in case both baits require different 3‐AT concentrations to block autoactivation of the HIS3 reporter gene, two sets of DOB‐L‐W‐H plates, each with the appropriate 3‐AT concentration, will have to be prepared.

### Confirming positive interactions and quantifying strength

17 Use positive colonies from DOB‐L‐W‐H ± 3‐AT medium to inoculate 3 mL of YPAD medium and incubate for 24 h at 28°C with shaking.
18 Isolate the AD‐prey plasmid using a yeast plasmid miniprep kit or any other appropriate procedure.
  We use a bacterial plasmid miniprep kit instead of a yeast plasmid miniprep kit by increasing lysis time from 5 to 10‐15 min. The yield is very low, but it is suitable for E. coli transformation. If this does not work for you, you can use 0.5 mm zirconium beads to break down the yeast and then use the bacterial miniprep kit.
19 Transform *Escherichia coli* with the isolated plasmid and reisolate it from the bacteria to sequence the prey ORF with the oligonucleotides GAL4AD‐F and pDEST‐R (Table 1).
20 Reintroduce this plasmid into the YM4271 yeast strain and repeat the mating with the bait strain, or directly introduce it into the bait strain by transformation. Plate the resulting yeast strain together with negative controls to confirm the interaction testing the 3‐AT concentration used in the screening and a narrow range of lower and higher levels.
  Scoring yeast growth for 7 days after plating cells will provide additional information about the strength of the interaction. Usually, faster growth suggests stronger interactions.
  On some occasions, the genetic background of the yeast cells (haploid versus diploid) may affect the interaction. Although mating (diploid background) detects fewer interactions compared to transformation (haploid background), the first option gives more reproducible results and is better suited for high‐throughput screens (Reece‐Hoyes & Walhout, 2012).

## AUTOMATED SCREENING WITH A LIQUID‐HANDLING ROBOT

**Figure 4:**
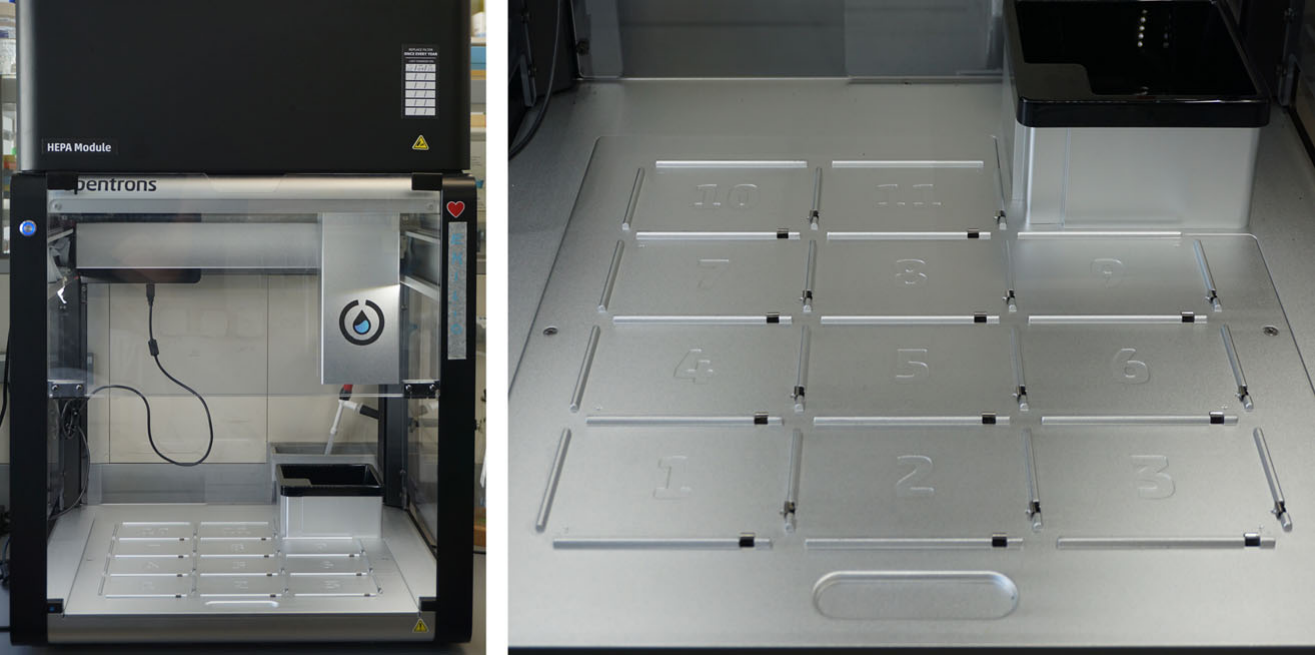
Opentrons 2 (OT‐2) pipetting robot (left). For this protocol, sterility is required; therefore, a HEPA module should be installed. OT‐2 features include 11 deck slots for accommodating labware in multiple configurations (right).

This alternate protocol allows you to partially automate the screening using an Opentrons 2 (OT‐2) robot and the entry LAP‐CellMediaInoculation‐OT2‐2.0.0 from the LAP Repository (Anhel et al., 2023). The flexibility of this open‐source technology has allowed us to adapt steps 13 to 15 to minimize manual labor and human error. OT‐2 features include 11 deck slots for accommodating labware in multiple configurations, a trash bin, and space for interchangeable single and 8‐channel pipettes (Fig. 4). As sterility is required for this protocol, a HEPA module should be installed.

This automation, as detailed below, is intended to generate 96‐well microtiter plates with aliquoted YPAD media (Day 1; step 13) or DOB‐L‐W (Day 4; step 15) media (number of plates to be specified by the researcher); aliquot the YPAD bait culture into each prey well (Day 2; step 14), and transfer a fraction of the mating mix to the DOB‐L‐W plates for diploid enrichment (Day 4; step 15).

### Hardware and Additional Materials

OT‐2 pipetting robot (Opentrons Labworks)
OT‐2 accessories:
  OT‐2 HEPA module (SKU 999‐00137 or 999‐00139)
  8‐channel pipette, min. P200 (we used OT‐2 8‐Channel GEN2 Pipette P300, SKU 999‐00006)
300‐µL universal tip pre‐sterile, racked (Neptune 2092.S, 63300520)
Reservoir
*You can use any sterilizable reservoir that fits into an Opentrons deck slot, even a 3D‐printed box or an empty rack of Neptune tips. We provide a custom labware definition recognized by the robot as a 96‐well reservoir*.

### Software

Microsoft Excel
Excel files: We provide ready‐to‐use files for each step of the Y1H screening protocol (see Supporting Information). These have been generated from the original Excel template found in the LAP repository.
Opentrons app (https://opentrons.com/ot‐app)
Customized labware definitions for the Neptune tip rack and reservoir (see Supporting Information)
Python script LAP‐CellMediaInoculation‐OT2‐2.0.0 from the LAP repository (Download from https://www.laprepo.com/protocol/cell‐inoculation‐in‐different‐media‐v2‐0‐0/)

### Script and robot preparation

1 If this is the first time you have connected your computer to OT‐2, you should have generated an OT public key. Follow the instructions on the official website: https://support.opentrons.com/s/article/Setting‐up‐SSH‐access‐to‐your‐OT‐2
2 Now, you can establish the connection between your computer and OT‐2 by transferring the public key. You can find the instructions at https://support.opentrons.com/s/article/Connecting‐to‐your‐OT‐2‐with‐SSH.
  From this point on, the steps include a Y1H screening adaptation of the OT‐2 media dispensing and culture inoculation protocol given in the LAP Repository. If required, a more detailed explanation can be found at dx.doi.org/10.17504/protocols.io.q26g7yb3kgwz/v3 (Anhel et al., 2024).
3 For the robot to recognize the Neptune tip rack and reservoir, you need to provide a custom labware definition to the Opentrons app. First, download the *neptune4_96_tiprack_300 ul.json* archive and upload it to the Labware library. Next, change the name of the archive to *1.json* and save it in a folder called *neptune4_96_tiprack_300 ul*. Finally, transfer the folder through the terminal with the following command:
scp ‐i [OT_key path] ‐r [path to neptune4_96_tiprack_300 ul fold] root@[IP_Robot]:/data/labware/v2/custom_definitions/custom_beta
  Repeat the process with the *mockindependanttubes3d_96_reservoir_1750 ul.json* archive and the following command:
scp ‐i [OT_key path] ‐r [path to mockindependanttubes3d_96_reservoir_1750 ul fold] root@[IP_Robot]:/data/labware/v2/custom_definitions/custom_beta
4 Download the provided Excel files and customize the sheets according to the guidelines in *InoculationInstructions.pdf* from *dx.doi.org/10.17504/protocols.io.q26g7yb3kgwz/v3*. You should define the number of samples you will be working with and change the type of plate, multichannel pipette, reservoir, or tip rack if they differ from the ones we listed. Save the resulting file as *VariablesPlateIncubation.xlsx* only.
  Always store the files with the name VariablesPlateIncubation.xlsx so they can be recognized by the Python script. We recommend saving them in different folders and renaming them (i.e., Day X or Step X).
5 Download the Python script LAP‐CellMediaInoculation‐OT2‐2.0.0. It can be found at https://www.laprepo.com/protocol/cell‐inoculation‐in‐different‐media‐v2‐0‐0/.
6 Rename the Python script according to the Excel file you want to send and upload it to the Opentrons app. A warning will appear during the protocol simulation because the script is not designed to read the Excel variable file from your computer but from the robot system.
7 The script is ready to read the corresponding *VariablesPlateIncubation.xlsx* file. Send it to the robot over the terminal:
scp ‐i [OT_key path] [VariablesPlateIncubation.xlsx path]
root@[IP_Robot]:/data/user_storage
  Note that if you are a macOS user, you should start the command with scp ‐O ‐i
  For finding your OT‐2's IP address, consult https://support.opentrons.com/s/article/Connecting‐to‐your‐OT‐2‐with‐SSH. You can find more information at https://support.opentrons.com/s/article/Copying‐files‐to‐and‐fromyour‐OT‐2‐with‐SCP.
8 Click on the protocol and select “Start setup” on OT‐2 where the file *VariablesPlateIncubation.xlsx* has been sent. Choose “To Setup” and the robot will show a simulation of the script with the required labware positions in the “Labware” tab and the reagents, with their corresponding volume, in the “Liquids” tab.
  The volume of the initial samples is established to be 90% of the maximum volume of the well, but this is only a recommendation. Just make sure that there is enough volume to transfer to all the final plates. It is always suggested to add more to account for the pipetting error.
9 Disinfect the robot's enclosure and the deck surface with 70% ethanol, and then gather the labware as the app shows.
10 Perform a labware offset, ensuring all the labware is calibrated correctly.
11 Close the door of the Opentrons and press the button “Start run.”

### Protocol execution

12 Day 1 (*VariablesPlateIncubation_day1.xlsx*; see Supporting Information): Pour the YPAD media and remove any lids or covers before starting the run. Using the OT‐2 8‐channel pipette, aliquot 125 µL of YPAD medium from the reservoir into each well of the 96‐well microtiter plates. Next, inoculate and incubate the library prey strains following Basic Protocol 2. In parallel, inoculate an Erlenmeyer containing 15 mL of YPAD medium/96‐well microtiter plate with a clump of bait cells from the DOB‐L plate and incubate for 24 h at 28°C with shaking (200 rpm).
  Labware: Sterile reservoirs and 96‐well microtiter plates are needed. The robot deck has 11 slots for positioning labware, two of which are occupied by the tip rack and the reservoir. As tip change is not required, the robot can be programmed to fill up to 9 plates in a single run.
  Media and disposables: We require 12‐15 mL YPAD medium/96‐well microtiter plate (200 mL for 15 microtiter plates), 12‐15 mL of YPAD bait culture/96‐well microtiter, and one column of tips from a Neptune tip rack. Remember to provide a little extra volume in the reservoir; otherwise, the robot may not be able to pipette it.
13 Day 2 (mating; *VariablesPlateIncubation_day2.xlsx*; see Supporting Information): Pour YPAD bait culture into the sterile reservoir and remove any lids or covers before starting the run. Using the OT‐2 8‐channel pipette, aliquot 100 µL to each well of the 96‐well microtiter plates from Day 1. Incubate as indicated in Basic Protocol 2.
  Labware: Sterile reservoir and inoculated 96‐well microtiter plates from Day 1
  As tip change is not required; the robot can be programmed to fill up to 9 plates in a single run.
  Media and disposables: One column of tips from the Neptune tip rack
14 Days 3 and 4 (diploid enrichment, plate preparation; *VariablesPlateIncubation_day3.xlsx*; see Supporting Information): Pour DOB‐L‐W media and remove any lids or covers before starting the run. Using the OT‐2 8‐channel pipette, aliquot 200 µL to each well of new 96‐well microtiter plates. This step can be performed to generate the final plates just before inoculation or on the previous day, and the plates are stored at 4°C.
  Labware: Sterile reservoir and 96‐well microtiter plates
  No tip change is required, so the robot can be programmed to fill up to 9 plates in a single run.
  Media and disposables: Around 19.2‐22 mL DOB‐L‐W/96‐well plate (300 mL for 15 × 96–well microtiter plates), 15 × 96–well microtiter plates, and one column of tips from a Neptune tip rack
  Remember to provide a little extra volume.
15 Day 4 (diploid enrichment, inoculation; *VariablesPlateIncubation_day4.xlsx*; see Supporting Information): The OT‐2 will resuspend the cells in the mating plates by pipetting 200 µL up and down three times before transferring 50 µL to the new 96‐well DOB‐L‐W microtiter plates prepared previously. Incubate as indicated in Basic Protocol 2.
  Labware: Mating and DOB‐L‐W 96‐well microtiter plates
  In this case, tips are changed after each sample transference. For the generation of a final plate, three slots are required (for the tip rack, mating plate, and plate with selective media); therefore, OT‐2 will produce a maximum of three plates per run.
  Media and disposables: One Neptune tip rack per plate
16 Continue with step 16 of Basic Protocol 2.

## REAGENTS AND SOLUTIONS

### 3‐amino‐1,2,4‐triazole stock solution (3‐AT)

Dissolve the appropriate amount of 3‐AT in water to obtain a 2 M solution (i.e., 4.2 g in 25 mL water). Sterilize by filtration.

Store this stock solution at −20°C. When required, the appropriate amount of the 3‐AT stock solution (depending on the desired final concentration) should be thawed and added to autoclaved minimal media (DOB‐L‐W‐H) once it has cooled down to 50‐60°C. Once thawed, the stock solution can be frozen again.

3‐AT is a competitive inhibitor of the product of the HIS3 reporter gene. This is a toxic substance and requires using personal protective equipment.

### DOB minimal media

Dissolve 26.71 g/L of dropout base medium (DOB; MP Biomedicals or bioWORLD) in deionized water with the appropriate amount of a complete supplement mixture (CSM; MP Biomedicals or bioWORLD) of amino acids lacking the one/s used for auxotrophic selection: CSM‐leucine (CSM‐L), CSM‐tryptophan (CSM‐W), CSM‐leucine‐tryptophan (CSM‐L‐W), or CSM‐leucine‐tryptophan‐histidine (CSM‐L‐W‐H). Add adenine hemisulfate (30 mg/L) and 20 g/L agar for solid media. Sterilize by autoclaving media for 10 min at 120°C.

We store media at RT (liquid) or at 4°C (solid) in darkness or subdued light.

The pH does not need to be adjusted, but it should be in the 5‐5.5 range.

All yeast strains mentioned here carry the ade2–101 mutation. If grown on media not supplemented with adenine (Sigma‐Aldrich), the colonies will develop a pink or red color due to the accumulation of a pigment derivative of 5‐aminoimidazole ribotide in the vacuoles (Smirnov et al., 1967; Weisman et al., 1987).

Autoclaving minimal media for longer times causes browning of the media, resulting in poor yeast growth.

We purchase DOB and CSMs either from MP Biomedicals or bioWORLD as it reduces labor and variability between media batches.

We usually use CSM‐L‐W‐H and 3‐AT for screening plates. Note that CSM‐leucine‐tryptophan‐histidine‐adenine (CSM‐L‐W‐H‐A) can also be used as more stringent media but be aware that this double selection system may be too restrictive and only reveal strong interactions. We have found that using CSM‐L‐W‐H‐A is equivalent to using ∼2.5 mM 3‐AT in the CSM‐L‐W‐H medium.

### Herring sperm DNA (HsDNA)

The product is supplied in a storage buffer (10 mM Tris·HCl (pH 7.5), 10 mM NaCl, and 1 mM EDTA) at a concentration of 10 mg/mL.

To avoid multiple freeze–thaw cycles and exposure to frequent temperature changes, preparation of small aliquots (1 mL) is recommended. Stocks should be kept at ‐20°C, whereas a working aliquot can be stored at 2‐5°C.

### YPAD medium

Dissolve 20 g/L peptone and 10 g/L yeast extract; adjust pH to 5.8 with HCl, top up to 950 mL with deionized water, and autoclave. Next, add adenine hemisulfate (30 mg/L) and 20 g/L agar for solid media. When autoclaved, add 50 mL of a 40% w/v glucose solution (2% final concentration).

We store media at RT (liquid) or at 4°C (solid) in darkness or subdued light.

### Yeast transformation solution (PATE)

Prepare and autoclave stock solutions of 50% w/v polyethylene glycol 4000 (PEG 4000; Merck), 1 M lithium acetate (LiAc), and 10 × Tris–EDTA (10 × TE: 100 mM Tris–HCl, 10 mM disodium EDTA, and pH 8.0 with HCl).

We always use PEG 4000 from Merck as we did not obtain transformants when we used the equivalent product from a different supplier.

To prepare the PATE working solution, mix the stock solutions to obtain 40% (w/v) PEG 4000, 0.1 M LiAc, and 1 × TE.

Once the working solution is prepared, it can be stored at RT.

## COMMENTARY

### Critical Parameters

**Figure 5:**
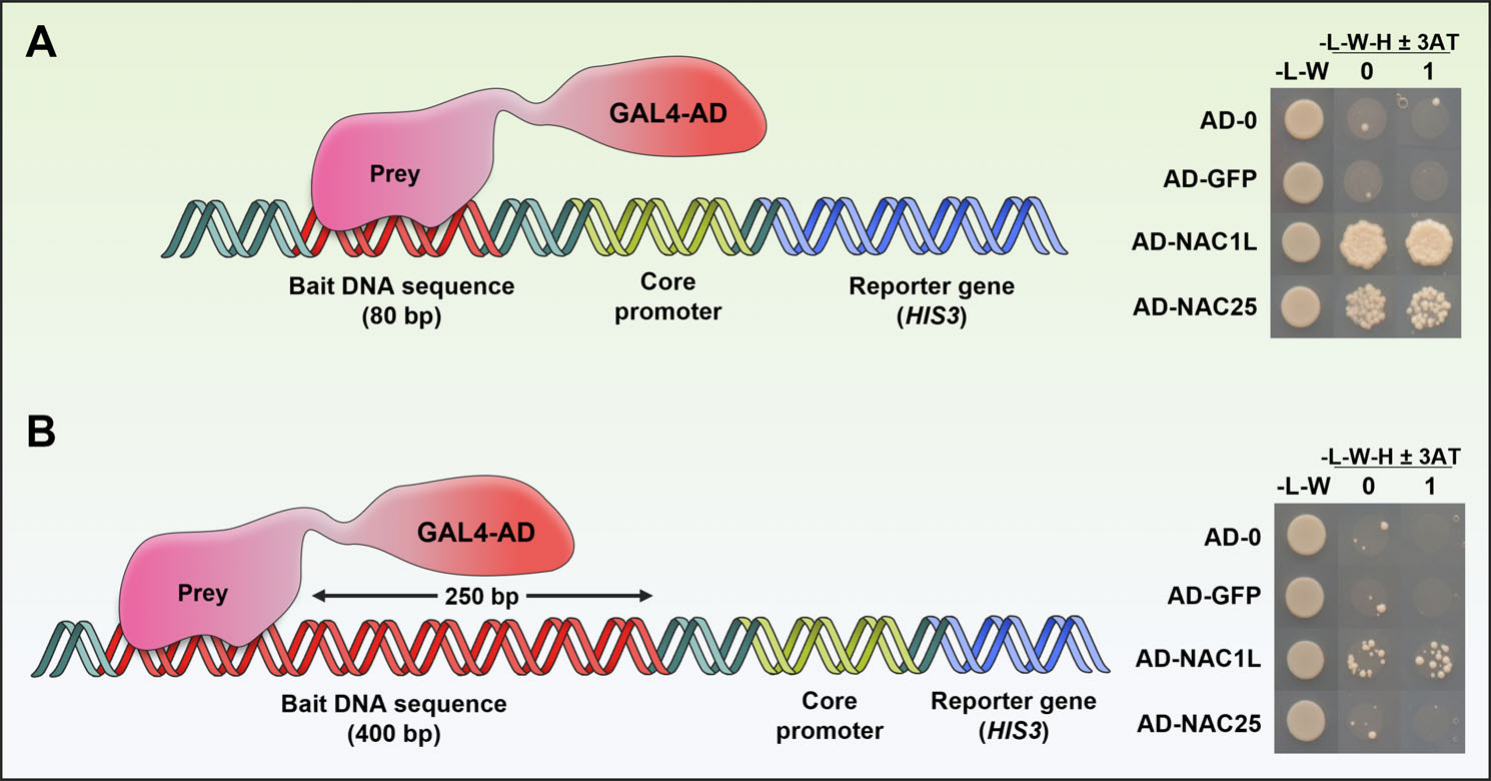
Effect of the distance between the bait DNA and the reporter gene. (**A**) An 80‐bp fragment of the *EXPA2* promoter bound by the NAC1L and NAC25 TFs activates *HIS3* reporter expression. (**B**) Yeast growth (*HIS3* activation) drops drastically when the 80‐bp fragment is shifted 250 bp further upstream of the reporter gene (unpub. observ.).

The main goal of a successful experimental procedure is to identify positive interactors able to bind to the DNA promoter sequence of your gene of interest. For that, the critical step is the selection of an optimal bait to perform screening. It is very important to select bait promoter fragments not much longer than 100 bp. *Saccharomyces cerevisiae* genome is more compact than those of plants and it is known that for upstream activating sequences (UAS) located over 300 bp upstream of a reporter gene, transcription initiates proximally to the UAS and competes with those derived from the reporter gene located downstream (Dobi & Winston, 2007). In our hands, the sensitivity of the assay is reduced considerably when the bound DNA sequence is too far away from the minimal promoter even if it is in the context of its own promoter (i.e., an 80‐bp sequence in a 400‐bp promoter fragment; Fig. 5). Moreover, using multimerized sequences tends to give higher backgrounds than using just one copy of the selected DNA sequence (L. Oñate‐Sánchez, unpub. observ.). The strength of this method is based on selecting fragments that are small to gain sensitivity, but large enough for a genomic context to exist. In this way, this approach can filter genetic redundancy based on the DNA‐binding specificity of *Arabidopsis* TFs, identifying only the binding of specific TFs within the same family. Some successful examples of the selection of DNA baits and identification of biologically relevant regulators done in our lab are given in Table 4.

**Table 4:** Biologically Relevant Regulators Identified/Confirmed by Y1H Screenings Carried Out with this Protocol

DNA bait	Conserved motif	Bait length (bp)	Proteins found	Family TFs^*a*^ in library ^*b*^	Ref.
IPS1	P1BS	50	PHR1	G2‐LIKE (44)	(Castrillo et al., 2011)
LIP1	L1‐box	50	AtML1	Homeobox (66)	(Castrillo et al., 2011; Rombolá‐Caldentey et al., 2014)
MAN7	G‐box	118	bZIP44	bZIP (63)	(Iglesias‐Fernández et al., 2013)
CathB3	G‐box	83	GBF1	bZIP (63)	(Iglesias‐Fernández et al., 2014)
NGA	TCP core BS	55	TCP1,2,3	TCP (23)	(Ballester et al., 2015)
EXPA2	NAC‐BS	80	NAC25, NAC1L	NAC (77)	(Sánchez‐Montesino et al., 2019)

^*a*^ TFs, transcription factors;

^*b*^ Number of TF family members present in the screened yeast library (Castrillo et al., 2011).

We have used this experimental procedure specifically with *A. thaliana*. When working with other plant species, the identification of six close orthologues for Basic Protocol 1 will depend on the availability of annotated genomes within the same family. However, even with a smaller number, you can still narrow down potential bait sequences by using a phylogenetic shadowing analysis. In addition, the online resources listed above (PlantCARE, PLACE, etc.) contain consensus TF binding motifs from several plant species, but you can also search for crop‐specific databases such as RiceTFtarget (Zhang et al., 2023). In this sense, for Basic Protocol 2, maize and rice TF libraries have been generated (Burdo et al., 2014; Xu et al., 2021). For other species, it may be necessary to use heterologous plant TF libraries (Parapunova et al., 2014) or cDNA libraries (Erffelinck et al., 2018; He et al., 2019); the latter approach is much less efficient for the identification of TFs because they represent only a small percentage of clones.

### Troubleshooting Table

Tables 5 and 6 delineate common problems that may be encountered in performing Basic Protocols 1, 2, and Alternate Protocol, with the possible causes and solutions we propose to address them.

**Table 5:** Troubleshooting Guide for Basic Protocol 1

Problem	Possible cause	Solution
No TF^*a*^ motifs or unreliable TF motifs found using online resources	Sometimes databases and tools can be somewhat confusing. Conserved motifs in your sequence may not yet be annotated as TF binding sites.	Perform bibliographic searches of known TF binding sites and search them in your sequence. Select your bait DNA sequence based on the most conserved blocks.

^*a*^ TF, transcription factor.

**Table 6:** Troubleshooting Guide for Basic Protocol 2 and Alternate Protocol

Problem	Possible cause	Solution
No yeast growth	Denatured alcohol used for replicator sterilization	Use absolute alcohol.
Red yeast appearance	Insufficient adenine in media for yeast growth	Add more adenine.
No perfect circles or formation of crescents in the solid plates	Insufficient resuspension of yeast sedimented in liquid media	Perform multiple rapid vertical and circular movements with the replicator until there is no sediment left in the wells.
Insufficient yeast growth	Insufficient diploid enrichment	Re‐enrich diploids from diploid plates. Please note that we have observed that fewer diploid cells are recovered when mating Y187 with YM4271 in Y1H compared to those obtained between pJ694 and YM4271 in Y2H experiments.
No positives in one week	Perhaps the 3‐ATa^ *a*^ concentration has not been properly titered	Re‐plate the enriched diploids on a new set of plates with a lower concentration of 3‐AT.

^*a*^ 3‐amino‐1,2,4‐triazole.

### Understanding Results

To illustrate how to carry out this method, we have reanalyzed and reassayed the *EXPA2* promoter, whose study led to uncovering a key role of the GA/DELLA‐NAC25/NAC1L‐EXPA2 network in regulating endosperm cell expansion (Sánchez‐Montesino et al., 2019).

**Figure 6:**
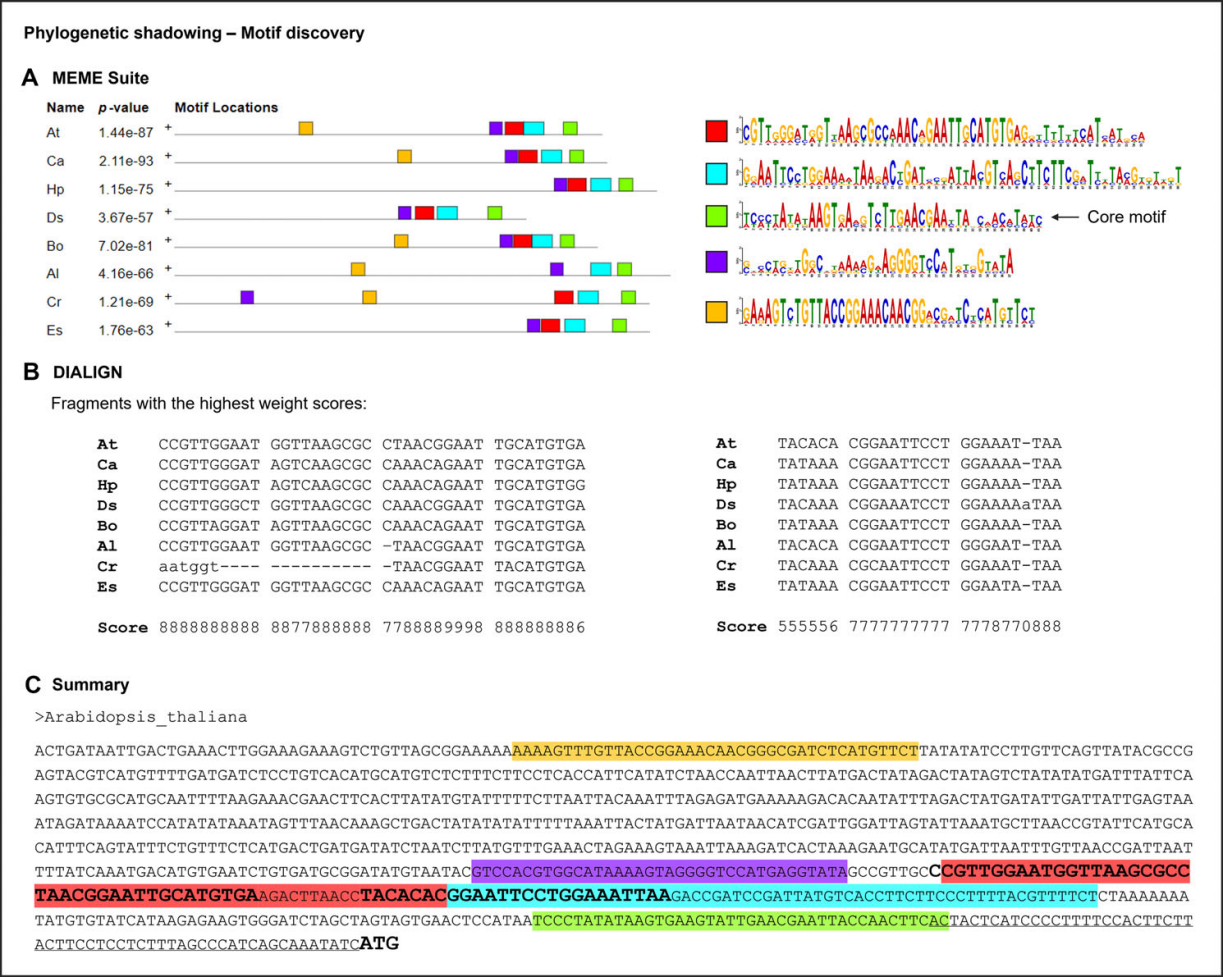
(**A**) Graphical representation of the location and sequence logo of the five most significant conserved motifs in the *EXPA2* promoter found by MEME Suite. (**B**) Aligned sequence fragments with the highest weight score in DIALIGN. Every position gets a value between 0 (no similarity) and 9 (maximum similarity). (**C**) Combined outputs of both online tools in the *Arabidopsis EXPA2* promoter: colored background from MEME Suite and bold type from DIALIGN.

To illustrate Basic Protocol 1, we used eight orthologues of the *Arabidopsis EXPA2* gene for the phylogenetic shadowing analysis. Promoter sequences of approximately 1.5 kb upstream of the predicted start of each ORF were compiled in FASTA format and uploaded to the MEME Suite and DIALIGN websites. The first tool (Fig. 6A) revealed two main regions of high conservation value across all the different species (red and cyan). You can see that there are some other highlighted areas in the sequence. However, one of them (light green) corresponds to the expected RNA polymerase core motif and should not be selected for this analysis, as it will not provide any useful information. The other two are much less promising, as they are absent (light orange) or have very few shared residues across the orthologues (purple). Similar to Meme Suite, DIALIGN alignments (Fig. 6B) showed two highly conserved motifs in the first 300 bp upstream of the start codon. With the combination of these tools, we were able to narrow down the potential bait sequence (Fig. 6C).

**Figure 7:**
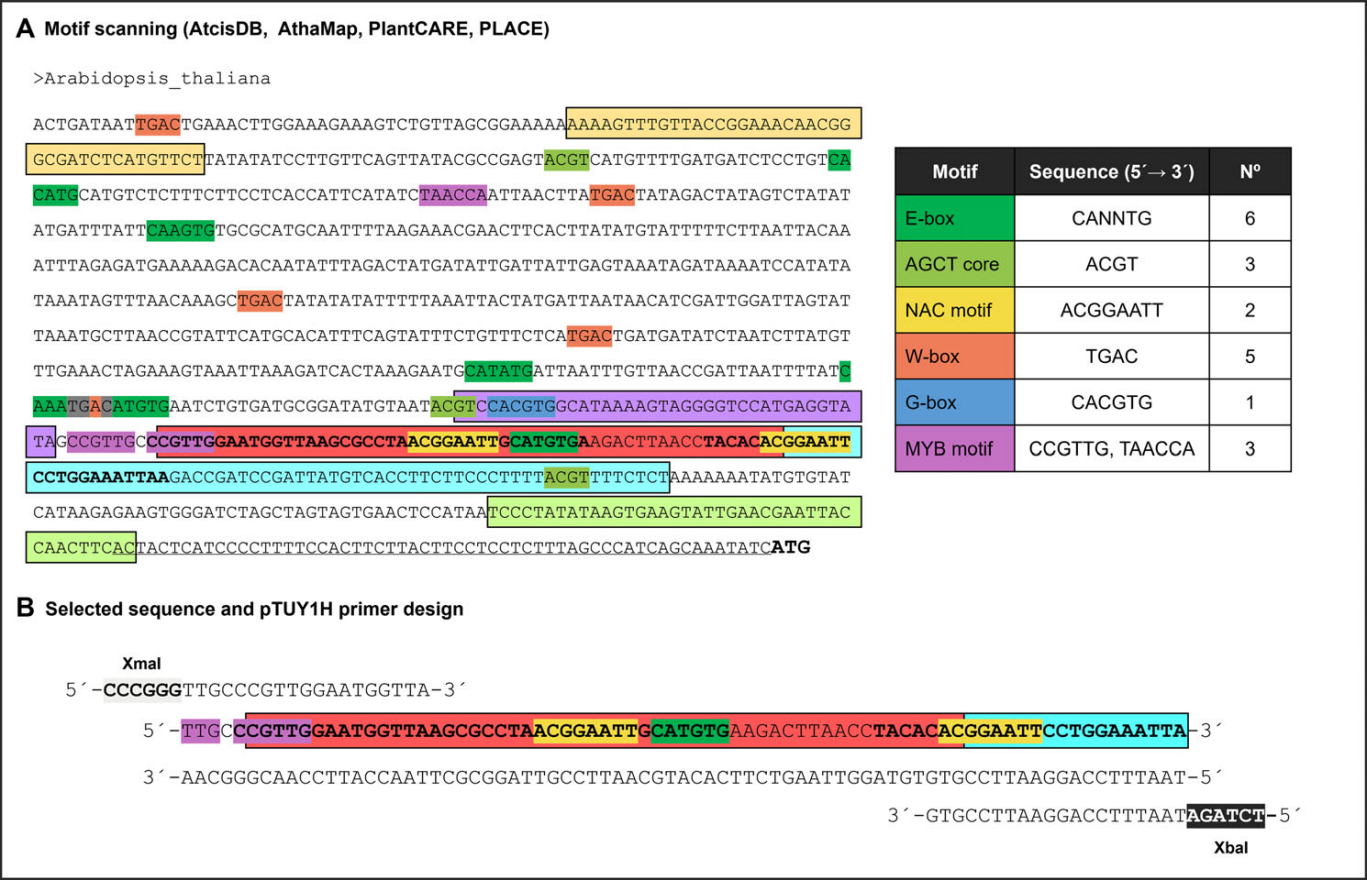
(**A**) Highlighted TF binding sites combined with the results of the phylogenetic shadowing analysis. (**B**) Selected DNA bait sequence and designed primers for its amplification according to the instructions provided in Basic Protocol 2.

The next step was to identify any annotated TF binding sites. After conducting a bibliographic search and collecting the results from various databases, we compiled the most significant motifs and confirmed that some of them overlapped with the phylogenetically conserved blocks (Fig. 7A). Taking all this into account, we designed the amplification primers so that our DNA bait contained the two highly conserved sequences (in the red and cyan regions), in which there were four annotated TF binding sites: a MYB motif, an E‐box, and two NAC motifs (Fig. 7B).

**Figure 8:**
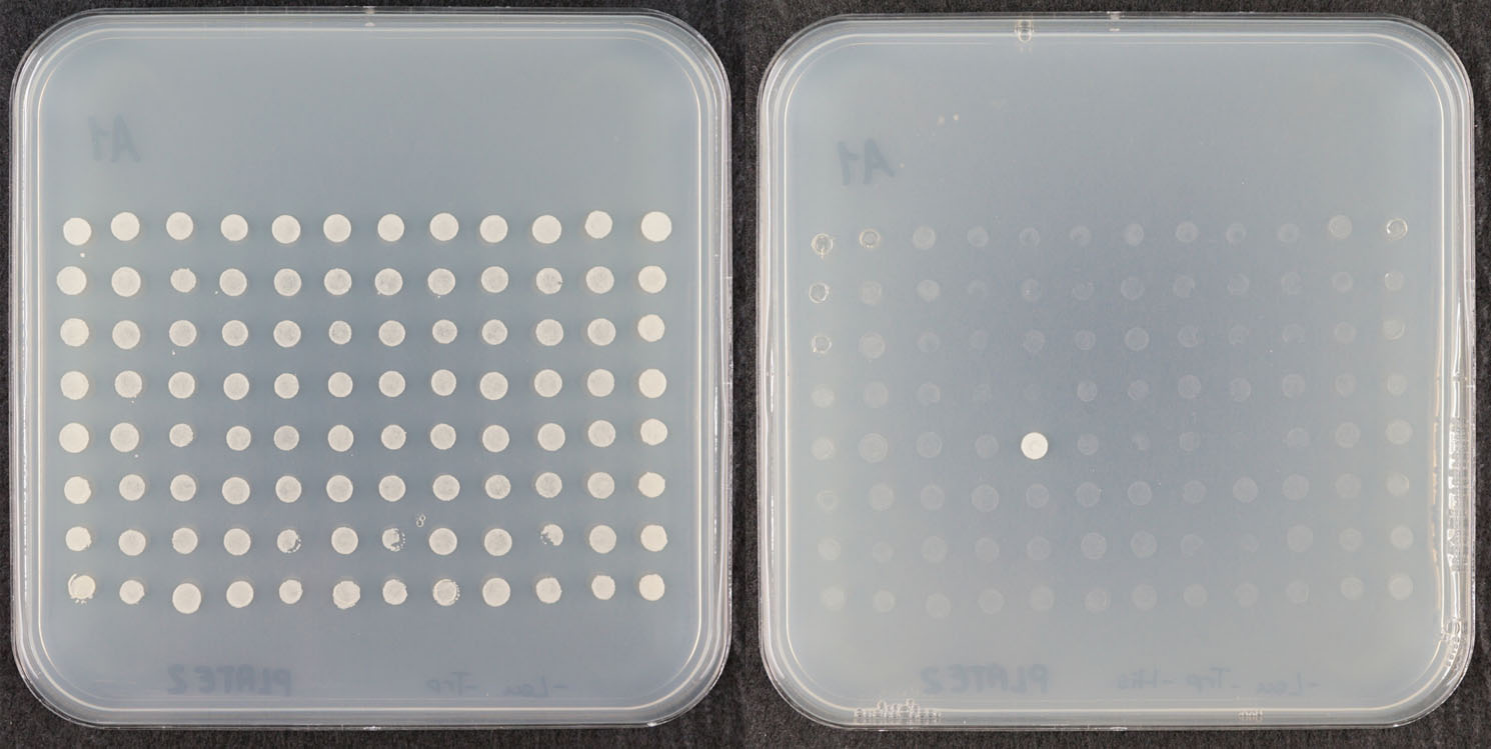
Result of a Y1H screening. Diploid plate 2 (left) confirms that the experiment has been carried out successfully. The selection plate (right) identifies if there is any protein capable of interacting with the promoter. 2E5 position was identified as NAC25 TF.

The 80‐bp fragment was amplified and cloned into the plasmid pTUY1H. Then, we titrated bait autoactivation of the *HIS3* reporter gene, setting 1 mM 3‐AT as the working concentration, as it is the lowest concentration that eliminates the yeast growth background after 1 week of scoring. We screened the bait with a 15 × 96–well microtiter plate prey library (Castrillo et al., 2011). As a result, only two yeast strains contained inserts able to activate *HIS3* under the control of the *EXPA2* 80‐bp fragment and grew on the 1 mM 3‐AT screening medium. We isolated and sequenced the corresponding GAL4AD‐TF plasmids, identifying two NAC TFs, NAC25 (At1g61110) and NAC1‐like (NAC1L; At3g12977; Sánchez‐Montesino et al., 2019). NAC25 was identified in position 2E5, so the diploid plate and selection plate 2 are shown as an example of a typical result (Fig. 8).

### Time Considerations

Basic Protocol 1 will take ∼1 day of work. If you complete the protocol with the optional analysis in planta, Basic Protocol 1 will take approximately 3 weeks to complete.

Basic Protocol 2 or Alternate Protocol can be finished in 10‐12 weeks. Time considerations and workflow are outlined in Table 7.

**Table 7:** Schematical Workflow for the Screening

**WEEK 1**			**WEEK 2**			
**Cloning**		**Yeast transformation**				
Generate bait construct with DNA sequence in pTUY1H	Generate negative controls in pDEST22	Streak yeast strains from frozen stock	Liquid culture YPAD	Mix yeast with carrier DNA, plasmid DNA, and PATE^*a*^ solution	Plating DOB‐L^*b*^ (pTUY1H) and DOB‐W^*c*^ (pDEST22)	Incubation
		72 h (weekend)	24 h			48‐72 h
**WEEK 3**				**WEEK 4**		**WEEK 5**
**Titrating autoactivation**						
Streak transformants and controls in DOB‐L and ‐W	Liquid culture YPAD	Mating	Diploid enrichment in DOB‐L‐W^*d*^	Plating DOB‐L‐W and DOB‐L‐W‐H^*e*^ ± 3‐AT^*f*^	Yeast growth scoring	
24 h	24 h	48 h	72 h (weekend)		7 days	
**WEEK 5**	**WEEK 6**			**WEEK 7**		**WEEK 8**
**Screening yeast arrayed libraries**						
Streak bait and refresh library in DOB‐L/‐W	Liquid culture YPAD	Mating	Diploid enrichment in DOB‐L‐W	Plating DOB‐L‐W DOB‐L‐W‐H ± 3‐AT	Yeast growth scoring	
72 h (weekend)	24 h	48 h	72 h (weekend)		7 days	
**WEEK 8**				**WEEK 9**	**WEEKS 10‐12**	
**Confirming positive interactions and quantifying strength**						
Inoculate YPAD with positive colonies from DOB‐L‐W‐H plate	Isolate the AD‐prey plasmid	Transform *E. coli*, reisolate, and sequence	Streak YM421 yeast strain	Yeast transformation	Repeat protocol from weeks 3 to 5	
24 h			72 h (weekend)			

^*a*^ PATE, yeast transformation solution;

^*b*^ DOB‐L, DOB‐leucine;

^*c*^ DOB‐W, DOB‐tryptophan;

^*d*^ DOB‐L‐W, DOB‐leucine‐tryptophan;

^*e*^ DOB‐L‐W‐H, DOB‐leucine‐tryptophan‐histidine;

^*f*^ 3‐AT, 3‐amino‐1,2,4‐triazole.

### Author Contributions

**Iris Fañanás‐Pueyo**: Conceptualization; methodology; visualization; writing—original draft; writing—review and editing. **Ana‐Mariya Anhel**: Methodology; software; writing—review and editing. **Ángel Goñi‐Moreno**: Methodology; software; supervision; writing—review and editing. **Luis Oñate‐Sánchez**: Conceptualization; methodology; resources; supervision; writing—review and editing. **Gerardo Carrera‐Castaño**: Conceptualization; supervision; visualization; writing—original draft; writing—review and editing.

### Conflict of Interest

The authors declare no conflict of interest.

## Supporting information

Ready‐to‐use files for each step of the Y1H screening protocol in Alternate Protocol:
neptune4_96_tiprack_300 ul.json
VariablesPlateIncubation_day1.xlsx

VariablesPlateIncubation_day2.xlsx

VariablesPlateIncubation_day3.xlsx

VariablesPlateIncubation_day4.xlsx

## Data Availability

Data sharing is not applicable to this article as no new data were created or analyzed in this study.
